# A corpus-based analysis of noun modifiers in L2 writing: The respective impact of L2 proficiency and L1 background

**DOI:** 10.1371/journal.pone.0320092

**Published:** 2025-03-18

**Authors:** Fatih Ünal Bozdağ, Junhua Mo, Gareth Morris

**Affiliations:** 1 Faculty of Science and Humanities, Osmaniye Korkut Ata University, Fakiusagi Mah, Merkez Osmaniye, Turkey; 2 School of Foreign Languages, Soochow University, Suzhou, China; 3 Centre for English Language Education, University of Nottingham, Ningbo, China; Faculty of Electrical Engineering, Computer Science and Information Technology Osijek, CROATIA

## Abstract

Complex noun phrases, as a distinctive feature of academic writing, pose an important learning task for L2 learners. Noun modifiers are the primary means of constructing complex noun phrases. Due to the development of natural language processing (NLP) technologies in recent years, noun phrase complexity, which is a micro-syntactic complexity indicator reflecting the complexity and diversity of clausal and phrasal structures, has emerged as an important research topic. This study applies Bayesian regression with informative priors to analyze the use of English noun modifiers by L2 learners of different proficiency levels and L1 backgrounds through the exploration of the EF Cambridge Open Language Database (EFCAMDAT) corpus. It finds that L2 proficiency has a significant impact on the development of noun phrase complexity in non-academic writing, while the influence of L1 background is observable but limited. It thus concludes that as second language proficiency increases, learners tend to converge towards a common grammatical competence that transcends their native linguistic frameworks.

## Introduction

Noun phrases (NP) are core syntactic structures that usually comprise nouns as their central elements [1]. In contemporary grammar, such structures typically function as the grammatical subject or object, the external complement positioned outside the verb phrase, although the semantic role associated with a noun phrase in a complement function can differ depending on the verb’s meaning [2]. Additionally, the internal organization of noun phrases is shaped not only by grammatical rules but also by communicative, pragmatic and cognitive factors, emphasizing the particularly complex interplay between syntax and semantics of noun phrases [3].

Noun phrases can be constructed by adding modifiers to the head noun. There are two types of modifiers: premodifiers and postmodifiers. Premodifiers include determiners, adjectives, possessives, nouns or participles that are used before the head noun, whereas postmodifiers include relative clauses, prepositional phrases, or possessor noun phrases following the head noun [4]. Premodification provides detailed information about the noun, enhancing the phrase’s economy and readability while also specifying and enriching the meaning of the head noun [5]. Similarly, postmodification further describes or qualifies the head noun, providing additional details or context. Both types of modification play crucial roles in expanding and refining the information conveyed by noun phrases.

Building on these concepts, both premodification and postmodification profoundly shape the structure and meaning of noun phrases. The complexity of noun phrases often depends on the nature and extent of their modifiers. These modifiers, whether lexical (e.g., adjectives and adverbs) or phrasal (e.g., prepositional phrases), significantly enhance the descriptive richness of noun phrases [6]. Taguchi et al. [7] indicate that “noun phrase modification (by attributive adjectives and post–noun-modifying prepositional phrases) had a tendency to contribute to essay quality” (pp. 428–429). Characterized by multiple premodifiers and postmodifiers, complex noun phrases are regarded as essential aspects of formal written language, highlighting the distinct characteristics of different discourse styles and genres [8]. Guillerit [9] further emphasizes the significance of complex noun phrases with multiple premodifiers and postmodifiers as crucial features of academic writing, contributing to linguistic enrichment and sophistication.

The modification of English noun phrases plays a crucial role in second language (L2) learning, reflecting learners’ understanding of grammatical structures and their ability to utilize linguistic elements. The analysis of English noun phrases has been frequently employed to gauge language proficiency based on the assumption that the complexity of learners’ noun phrases serves as an indicator of language proficiency [4]. Despite some confirmations, such an assumption needs more testing, preferably with larger amounts of learner data and more advanced statistical analyses. This study takes a novel corpus-based approach to investigating noun modifiers in L2 writing. Specifically, it makes a Bayesian analysis of the use of English noun modifiers by L2 learners of different proficiency levels and first language (L1) backgrounds by probing into the EF Cambridge Open Language Database (EFCAMDAT) corpus, which is a comprehensive collection of written and spoken texts from L2 learners and an open database for L2 research. The Bayesian framework is chosen for its ability to effectively model the inherent uncertainty and variability present in complex educational datasets like EFCAMDAT. By incorporating prior knowledge and updating beliefs based on observed data, the Bayesian approach is well-suited for handling uncertainty and allows for a comprehensive analysis of such data. This robust method provides a way to quantify evidence supporting hypotheses about how learners with different proficiency levels and linguistic backgrounds use various types of noun modifiers.

### Literature review

This review examines three critical domains in L2 noun modifier research: the developmental trajectory of noun phrase complexity, the relationship between noun modifiers and L2 proficiency, and the influence of L1 background on noun modifier usage. The synthesis of existing literature identifies significant research gaps that the present study addresses.

### NP complexity development

Biber et al. [8] argue that complex NPs are more appropriate indicators for measuring the grammatical complexity of academic writing than embedded clauses. Based on the analysis of a large-scale academic text corpus, they hypothesized five developmental stages for complexity features in academic writing, including 11 types of noun modifiers at phrasal and clausal levels. The first stage does not involve the complexity of noun phrase structures, mainly consisting of a limited number of object clauses controlled by common verbs. The second stage begins to show simple noun modifier features modified by adjectives and participle premodifiers. The third stage develops into more complex noun modifier patterns, such as nouns as premodifiers, prepositional phrases with specific meanings as postmodifiers of nouns, and relative clauses. The fourth stage sees the emergence of non-restrictive clauses modifying nouns, prepositional phrases with abstract meanings as postmodifiers of nouns, and adjective-noun sequences as premodifiers. The final stage includes appositive noun phrases and complement clauses as noun modifiers, and multiple phrase embeddings.

The validity of these developmental stages has been the subject of considerable debate. For example, Yang [10] pointed out that these hypotheses were not based on developmental research but on corpus studies of syntactic complexity characteristics in two different language domains (speech and writing). In addition, the data collected by Biber et al. [8] were from proficient L1 writers and speakers, not second language learners (i.e., the target group for their claims). Biber et al. [11], however, emphasized in their response that the main purpose of their 2011 study was to provide a comprehensive description of the grammatical features commonly found in advanced academic writing. They proposed that the production of academic discourse, especially that containing complex noun phrase structures, was a challenge for all students and professionals, regardless of their L1 background.

### Noun modifiers and L2 proficiency

Noun modifiers can serve as an effective indicator of second language writing proficiency. Among these, prenominal modifiers, participial postmodifiers, attributive adjectives, and prepositional postmodifiers are the most commonly measured structures [12]. Parkinson and Musgrave [13] investigated the noun phrase use of two groups of L2 learners and found that less proficient L2 learners used much more attributive adjectives, but much less premodifying nouns and postmodifying prepositional phrases than learners at the higher proficiency level.

Several studies have focused on Chinese learners of English. Liu and Li [14] found that even the noun phrase complexity of Chinese English learners at the postgraduate level was significantly lower than that of published corpora. Wang and Slater [15] discovered that there was a significant difference in the use of complex noun phrases between the writings of Chinese students and proficient users of English. Wang and Beckett [16] also noted significant differences in the noun phrase use between Chinese students and more proficient English users. Specifically, Chinese students tended to use more premodifiers for nouns but fewer postmodifiers, among which the use of postmodifying prepositional phrases was the most striking difference between these two groups.

At more advanced levels, Ansarifar et al. [17] uncovered that second language master of arts (MA) abstract authors differed significantly from the expert writers in the use of four types of modifiers: premodifying nouns, -*ed* participles as postmodifiers, adjective-noun sequences as premodifiers, and multiple prepositional phrases as noun postmodifiers. Doctoral writers, however, were similar to disciplinary experts, differing only in the use of multiple prepositional phrases as noun postmodifiers.

Further research has corroborated these findings. Lan and Sun [18] examined the complexity of NPs in the writing of first-year Chinese students and found that the higher the writing proficiency of the students, the greater the proportion of noun modifiers they used in writing. They also noted that first-year Chinese college students used fewer noun modifiers in writing compared to authors of academic journal articles, with the four types of modifiers accounting for most of this difference: adjectives, nouns as modifiers, prepositional phrases, and appositive noun phrases. Lan et al. [19] also found a significant association between L2 writing proficiency and NP complexity. Specifically, high-proficiency students used more attributive adjectives and relative clauses, while low-proficiency students used more premodifying nouns and prepositional phrases (of). By examining the complexity of noun phrases produced by advanced Chinese learners in integrated writing, Xu [20] detected a moderate positive correlation between the use of complex nouns in these learners’ writing and the scores given by expert raters. Lan et al. [21] found that L1 essays exhibited a wider variety of noun phrase patterns, while L2 essays tended to rely more on compressed noun phrases, which are a characteristic of advanced academic writing. They suggested that L2 students might have used formulaic sequences more frequently, contributing to the higher complexity of compressed noun phrases in their writing.

Studies on other L2 populations have shown similar patterns. Through observing the complexity of noun phrases in writing samples of young Spanish students, Díez-Bedmar and Pérez-Paredes [22] reported that as proficiency increased, so did the use of nouns as premodifiers and prepositional phrases as postmodifiers. Sarte and Gnevsheva [23] found that L2 writers with lowest proficiency used fewer noun modifiers than higher proficiency groups at all stages of phrasal complexity, thus confirming that noun phrasal complexity can discriminate L2 writing proficiency.

### Noun modifiers and L1 background

The influence of L1 background has been widely found in L2 use of noun modifiers. Chan [24] made a contrastive analysis of noun phrases in English and Chinese and suggested that cross-linguistic differences were partly responsible for English structural problems encountered by Chinese ESL students in Hong Kong. Carrió Pastor [25] reported a mother tongue interference in Spanish students’ translation of English premodified complex noun phrases. Albelihi and Lan [26] investigated the influence of language background on the use of NP complexity in the introductions of English dissertations written by L1 English and L1 Arabic students. They found that language background significantly influences the use of four types of noun modifiers: premodifying nouns, prepositional phrases (other), prepositions followed by -*ing* clauses, and infinitive clauses. Haryanto and Tedjasuksmana [27] discovered that cross-linguistic differences were one of the reasons that Indonesian students commit errors in the use of English relative clauses as postmodifiers for noun phrases in their thesis writing. Li and Tang [28] reported an L1 influence on L2 writing as they found that low complexity NPs (e.g., relative clause) were lacking in English writing of Chinese postgraduates because it does not exist in Mandarin. In contrast, English NPs (e.g., attributive adjective), which could equate to Mandarin NP structures, were used more frequently.

### Research gaps

Three research gaps can be identified in the existing studies on L2 use of noun modifiers. Firstly, given that the existing studies are mainly focused on academic writing, it remains unknown whether their findings are applicable to general writing. Existing studies underscore the prominent role of writing proficiency as a key factor influencing the development of NP complexity and modification. However, the notion of complex phrases is often attributed almost exclusively to academic discourse, and the term of complexity remains vaguely defined beyond the length of structures. While noun phrases in learner language undoubtedly reflect the progression of syntactic skills and the ability to construct intricate phrases—crucial for academic writing—it is essential to recognize that different types of modification necessitate a comprehensive understanding of interconnected grammatical concepts. This represents an expected developmental progression that is not necessarily limited to academic writing skills. Additionally, although existing studies have detected the influence of L1 background on L2 use of noun modifiers, it remains unclear how this influence changes with the improvement of L2 proficiency. Finally, determining which has a greater impact, second language proficiency or first language background, necessitates further investigation. Therefore, this study attempts to fill in these gaps by investigating the use of English noun modifiers by learners of different L2 proficiency levels and L1 backgrounds in non-academic writing settings.

### Research aims

This study employed a mixed-methods research design, combining quantitative corpus analysis with qualitative interpretation of linguistic patterns. The research framework examined two main factors: L2 proficiency levels (B1, B2, C1) and L1 backgrounds (10 different language groups) as independent variables, with the frequency and distribution of 10 types of noun modifiers as dependent variables. Text type and writing context within the EFCAMDAT corpus were maintained as control variables to ensure consistency. The quantitative component used Bayesian statistical analysis to examine frequency distributions and patterns, while the qualitative component analyzed how different learner groups used noun modifiers. This mixed-methods approach provided both statistical evidence and meaningful insights into how learners use noun modifiers across different proficiency levels and L1 backgrounds.

### Research questions

This study intends to answer three research questions.

  RQ1. How are different types of noun modifiers used by L2 learners of different proficiency levels?

  RQ2. How are different types of noun modifiers used by L2 learners of different L1 backgrounds?

  RQ3. How do the frequency and diversity of premodification and postmodification strategies evolve with the development of L2 proficiency?

## Methodology

This section delineates the research design and analytical framework employed to investigate noun modifier usage patterns. Specifically, it details the corpus selection criteria, data extraction procedures, and the implementation of Bayesian statistical analysis.

### Learner corpora

This study leverages the EFCAMDAT corpus, which is a comprehensive collection of 1,180,310 texts written by 174,743 learners of diverse nationalities [29]. Specifically, it utilizes the pre-processed version of the corpus prepared by Shatz [30], which includes cleaned texts. This dataset offers a wide range of English proficiency levels measured by the Common European Framework of Reference for Languages (CEFR), which is a widely recognized framework used to describe language proficiency levels in a structured manner across Europe and beyond. CEFR provides a common basis for comparing language skills, dividing them into six levels: A1, A2, B1, B2, C1, and C2, ranging from beginner (A1) to proficient (C2). It is important to note that while noun phrase complexity can serve as one indicator of writing proficiency, in this study, proficiency levels were determined through the EFCAMDAT corpus’ comprehensive assessment framework, which evaluates multiple aspects of language competence. Therefore, the analysis examines the relationship between these pre-determined proficiency levels and noun modifier usage patterns, rather than using noun phrase complexity to define proficiency itself.

For the current study, however, the corpus was subset into three corpora, each representing three proficiency levels, including B1, B2, and C1. The data for lower levels were excluded due to learners’ likelihood of utilizing highly formulaic constructions, both due to their proficiency levels and the nature of topics to which they were asked to respond. These include, for example, greeting someone, introducing yourself, and writing an e-mail. Additionally, mastering certain noun phrase modifications presumes mastering related grammatical topics which are not covered in lower levels’ curricula following the CEFR guideline.

Regarding nationality, texts from the 10 nationalities with most texts were kept: Saudi Arabian, French, German, Italian, Japanese, Chinese (including Taiwanese), Brazilian, Russian, Mexican, and Turkish. Following that, the subcorpus of three proficiency levels was divided into ten sub-corpora, each representing different first languages regardless of proficiency levels.

### Data retrieval and analysis

Extraction of noun phrases with modifications was handled via a custom Python script employing Spacy NLP [31], combining dependency parsing and Part-of-Speech tagging. Although this study parsed the cleaned version of the EFCAMDAT corpus provided by Shatz [30], to further enhance data quality, pre-and post-processing was performed to remove non-word characters and any remaining non-English words. It is necessary to point out that the accuracy of tagging and classification of structures depends on the overall success of the script and model. Though over a highly structured dataset it achieved roughly over 90% accuracy, the figures may change over learner data.

When classifying noun modifiers, this study mainly followed Lan et al.’s [21] suit to adopt Biber et al.’s [8] scheme of classifying noun modifiers into 11 types. This study, however, adopted 10 of them except for appositive noun phrases (e.g., ‘the two leaders, Smith and Jones’) in that the automatic extraction and annotation of appositive noun phrases returned poor results in terms of classification accuracy. The remaining 10 types of noun modifiers investigated in this study are further classified by positions and types and illustrated with examples in Table 1.

**Table 1:** Classification of noun modifiers by position and type with examples.

Noun modifier	Position	Type	Example
Attributive adjectives	pre	phrasal	philosophical position
Premodifying nouns	pre	phrasal	language barrier
Relative clauses	post	clausal	results that show differences
*-ing* clauses	post	clausal	a study examining differences
*-ed* clauses	post	clausal	a policy created by a coalition
Prepositional phrases (of)	post	phrasal	the methodology of the experiment
Prepositional phrases (other)	post	phrasal	several groups in the study
Prepositions + *ing* clauses	post	mixed	the idea of reforming the regulations
Noun complement clauses (that)	post	clausal	the proposition that knowledge is justiﬁed
Infinitive clauses	post	clausal	a massive plan to complete

**Fig 1:**
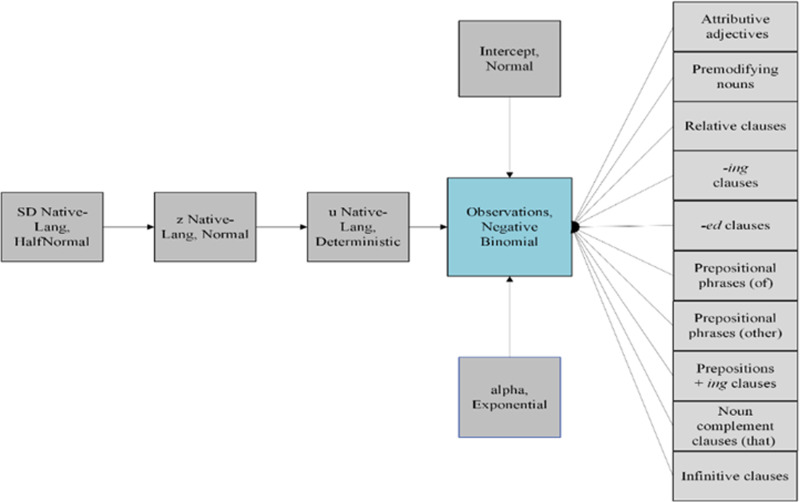
Visualization of the Bayesian regression model.

This study unfolded over three complementary statistics: relative frequency analysis, Z-score normalization, and regression analysis. First, relative frequencies were calculated within each subcorpus to observe the prevalence of specific modification types relative to their total usage. Next, raw frequencies were normalized with Z-score to indicate how far and in what direction frequencies per CEFR level and per first language deviated from the mean, with positive values suggesting above-average usage and negative values suggesting below-average usage relative to the overall figure in three corpora. Finally, a Bayesian negative binomial regression model was structured to investigate the effects of various noun modifiers on the frequency of their usage across different CEFR levels and among learners with different native languages (see Fig 1).

To elaborate, the priors for prepositions + *ing* clauses were set with mean values progressing from 0.05 at CEFR level B1 to 0.25 at C1, corresponding sigma values tapering from 0.2 to 0.1, reflecting anticipated advances in grammatical sophistication with increasing proficiency. Similarly, relative clauses were modeled with means escalating from 0.1 at B1 to 0.4 at C1, capturing expected improvements in the usage of complex syntactic structures. Prepositional phrases and infinitive clauses followed comparable trajectories, indicating a progression in handling these structures more adeptly at higher proficiency levels. Additionally, noun modifiers such as noun complement clauses (that) and descriptors like attributive adjectives were also modeled with increasing means from B1 to C1, supporting the notion of better descriptive and syntactic capabilities in advanced stages of language acquisition.

These assumptions were critical for the validity and reliability of the findings derived from the model, highlighting the interconnectedness of linguistic development, proficiency level, and native language influence in language acquisition research. The derived posterior proficiency effects determined the extent to which the empirical frequency data corresponded with or deviated from the prior expectations grounded in the CEFR guidelines. More importantly, this Bayesian approach facilitated an analysis that merged data-driven insights and theoretical constraints from pedagogical practices, offering a richer and more detailed perspective on noun modifier use grounded in CEFR-guided language acquisition.

## Results

The results are presented in three subsections aligned with the research questions: the distribution of noun modifiers across proficiency levels, patterns of modifier usage among different L1 groups, and the developmental progression of premodification and postmodification strategies. Each subsection presents quantitative analyses supported by statistical evidence. The findings are presented according to the three research questions that guided this study.

### Use of noun modifiers by L2 learners of different proficiency levels

Table 2 presents a detailed analysis of the distribution of noun modifiers across three proficiency CEFR levels: B1, B2, and C1. The table includes 10 types of noun modifiers and their respective metrics, such as raw frequency, relative frequency and Z-score. Raw frequency is provided to demonstrate the presence of noun modifiers. It cannot be used for direct comparison between learners at different levels in that the sizes of the three learner corpora are not identical. Instead, relative frequency and Z-score are metrics that can be used for cross-comparison of subcorpora, the feasibility of which is ensured by the Bayesian analysis adopted by the present study.

**Table 2:** Distribution of noun modifiers across different proficiency levels.

Noun modifiers	Metrics	CEFR levels		
		B1	B2	C1
Attributive adjectives	Raw frequency	221324	104393	45647
	Relative frequency	0.370	0.389	0.429
	Z-score	2.28	2.45	2.62
Premodifying nouns	Raw frequency	142613	52523	17823
	Relative frequency	0.238	0.196	0.167
	Z-score	1.17	0.81	0.54
Relative clauses	Raw frequency	39157	19073	8068
	Relative frequency	0.065	0.071	0.076
	Z-score	−0.29	−0.25	−0.19
-*ing* clauses	Raw frequency	2980	2229	842
	Relative frequency	0.005	0.008	0.008
	Z-score	−0.80	−0.78	−0.73
-*ed* clauses	Raw frequency	11181	3335	1459
	Relative frequency	0.019	0.012	0.014
	Z-score	−0.69	−0.74	−0.69
Prepositional phrases (of)	Raw frequency	66210	31397	13041
	Relative frequency	0.111	0.117	0.122
	Z-score	0.09	0.14	0.18
Prepositional phrases (other)	Raw frequency	101876	46275	16586
	Relative frequency	0.170	0.172	0.156
	Z-score	0.59	0.61	0.44
Prepositions + *ing* clauses	Raw frequency	3472	3436	1141
	Relative frequency	0.006	0.013	0.011
	Z-score	−0.80	−0.74	−0.71
Noun complement clauses (that)	Raw frequency	837	675	284
	Relative frequency	0.001	0.003	0.003
	Z-score	−0.83	−0.83	−0.78
Infinitive clauses	Raw frequency	8363	5069	1569
	Relative frequency	0.014	0.019	0.015
	Z-score	−0.73	−0.69	−0.68

Table 2 shows that of the 10 types of noun modifiers, attributive adjectives consistently demonstrate the highest relative frequency across CEFR levels, rising from 0.370 at B1 to 0.429 at C1. This increase suggests that as language proficiency improves, learners employ a wider range of vocabulary for more precise descriptive language.

Relative clauses also increase from 0.065 at B1 level to 0.076 at C1, indicating development of complex sentence structures. Among postnominal clausal modifiers, -*ed* clauses decrease from 0.019 to 0.014, while infinitive clauses show a slight increase from 0.014 to 0.015 at C1. Premodifying nouns show a decreasing pattern from B1 (0.238) to C1 (0.167), suggesting diversification in modification strategies. Postnominal prepositional phrases fluctuate across CEFR levels, moving from 0.281 at B1 (0.111 for the “of” type + 0.170 for the “other” type) to 0.289 at B2 (0.117 for the “of” type +  the 0.172 for the “other” type), then slightly decreasing to 0.278 at C1 (0.122 for the “of” type +  0.156 for the “other” type).

Prepositions followed by -*ing* clauses maintain a consistently low frequency, increasing from 0.006 at B1 to 0.011 at C1, with a peak of 0.013 at B2. Noun complement clauses (that) show very low usage but increase from 0.001 at B1 to 0.003 at C1. Overall, the analysis reveals a progression characterized by increased reliance on attributive adjectives and gradual integration of more complex modifiers, reflecting development from fundamental to more sophisticated syntactic structures.

Z-scores in Table 2 describe a similar picture to relative frequencies. Attributive adjectives consistently acquire positive Z-scores across all proficiency levels, with a progressive increase from B1 (Z =  2.283) to C1 (Z =  2.620), suggesting increased usage as proficiency advances. Conversely, all postnominal clausal modifiers (-*ed* clauses, infinitive clauses, -*ing* clauses, noun complement clauses, and relative clauses) consistently exhibit negative Z-scores across proficiency levels, indicating their less frequent usage than other structures.

Premodifying nouns, starting with a positive Z-score at B1 (Z =  1.170), decrease to a lower score at C1 (Z =  0.537), reflecting a shift in noun phrase construction complexity at higher proficiency levels. Postnominal phrasal modifiers show varying trends: prepositional phrases (of) display a slight increase in Z-scores from B1 to C1, while other prepositional phrases decrease from 0.59 at B1 to 0.44 at C1. Prepositional -*ing* clauses consistently register negative Z-scores, with a minor decrease in magnitude from −0.796 at B1 to −0.711 at C1.

A comparative analysis of relative frequency and Z-score trends reveals valuable insights into noun phrase modification acquisition. For example, attributive adjectives increase in both relative frequency (0.370 to 0.429) and Z-scores (2.283 to 2.620), indicating their growing prominence in learner language. Conversely, structures like -*ed* clauses and prepositional -*ing* clauses maintain low relative frequencies and negative Z-scores, suggesting persistent limited use despite proficiency advancement. These patterns highlight how certain grammatical structures become more standard with increased proficiency while others remain challenging.

### Use of noun modifiers by L2 learners from different L1 backgrounds

Table 3 presents a detailed analysis of the distribution of noun modifiers among L2 learners from 10 different first languages. The table includes 10 types of noun modifiers and their respective metrics, such as raw frequency, relative frequency and Z-score. As explained in Section 4.1, raw frequency is provided to show the presence of noun modifiers, whereas relative frequency and Z-score are used for direct comparisons.

**Table 3:** Distribution of noun modifiers among L2 learners from different L1 backgrounds.

Noun modifiers	Metrics	L1 backgrounds									
		AR	FR	DE	IT	JP	CN	PT	RU	ES	TR
Attributive adjectives	Raw F	7.898	22.198	45.517	29.55	11.429	65.404	116.842	45.238	21.624	5.664
	Rel. F	0.364	0.377	0.396	0.386	0.382	0.388	0.372	0.4	0.36	0.379
	Z-score	2.23	2.38	2.42	2.45	2.33	2.34	2.36	2.43	2.29	2.26
Premodifying nouns	Raw F	5.373	11.84	23.748	14.939	7.273	42.966	65.796	24.038	13.108	3.878
	Rel. F	0.248	0.201	0.206	0.195	0.243	0.255	0.209	0.212	0.218	0.259
	Z-score	1.25	0.87	0.87	0.82	1.18	1.26	0.95	0.91	1.04	1.29
Relative clauses	Raw F	1.395	3.942	7.102	5.414	1.835	10.438	24.662	5.974	4.672	864
	Rel. F	0.064	0.067	0.062	0.071	0.061	0.062	0.079	0.053	0.078	0.058
	Z-score	−0.3	−0.29	−0.31	−0.25	−0.32	−0.31	−0.19	−0.38	−0.2	−0.34
-*ing* clauses	Raw F	120	352	663	578	166	861	2.225	605	417	64
	Rel. F	0.006	0.006	0.006	0.008	0.006	0.005	0.007	0.005	0.007	0.004
	Z-score	−0.8	−0.81	−0.77	−0.79	−0.78	−0.77	−0.81	−0.77	−0.82	−0.78
-*ed* clauses	Raw F	306	1.226	1.36	1.731	403	2.52	6.097	1.179	994	159
	Rel. F	0.014	0.021	0.012	0.023	0.013	0.015	0.019	0.01	0.017	0.011
	Z-score	−0.72	−0.68	−0.72	−0.66	−0.71	−0.69	−0.7	−0.73	−0.73	−0.72
Prepositional phrases (of)	Raw F	2.171	7.374	12.677	9.575	3.436	16.928	36.023	14.019	6.871	1.574
	Rel. F	0.1	0.125	0.11	0.125	0.115	0.1	0.115	0.124	0.114	0.105
	Z-score	0	0.22	0.08	0.22	0.12	0	0.13	0.19	0.13	0.04
Prepositional phrases (other)	Raw F	3.852	10.491	20.935	12.696	4.665	25.009	54.655	19.288	10.699	2.447
	Rel. F	0.178	0.178	0.182	0.166	0.156	0.148	0.174	0.17	0.178	0.164
	Z-score	0.66	0.67	0.67	0.56	0.46	0.39	0.64	0.57	0.69	0.52
Prepositions + *ing* clauses	Raw F	215	484	1.128	627	263	1.477	2.192	1.089	460	114
	Rel. F	0.01	0.008	0.01	0.008	0.009	0.009	0.007	0.01	0.008	0.008
	Z-score	−0.76	−0.79	−0.74	−0.79	−0.75	−0.74	−0.81	−0.73	−0.81	−0.75
Noun complement clauses (that)	Raw F	20	95	286	103	69	405	481	221	80	36
	Rel. F	0.001	0.002	0.002	0.001	0.002	0.002	0.002	0.002	0.001	0.002
	Z-score	−0.84	−0.85	−0.8	−0.84	−0.81	−0.79	−0.85	−0.79	−0.87	−0.79
Infinitive clauses	Raw F	322	927	1.601	1.25	366	2.605	5.096	1.584	1.102	148
	Rel. F	0.015	0.016	0.014	0.016	0.012	0.015	0.016	0.014	0.018	0.01
	Z-score	−0.72	−0.73	−0.71	−0.72	−0.72	−0.69	−0.73	−0.7	−0.72	−0.73

AR stands for Arabic while FR for French, DE for German, IT for Italian, JA for Japanese, ZH for Chinese (Mandarin), PT for Portuguese, RU for Russian, ES for Spanish and TR for Turkish.

F is the abbreviation of frequency while Rel. that of relative.

Table 3 reveals consistent preference for attributive adjectives across all L2 learners, with relative frequencies ranging from 0.364 (Arabic) to 0.400 (Russian). Their Z-scores are consistently positive across all language backgrounds, with Italian speakers showing the highest (Z =  2.45) and Turkish speakers the lowest but still notable preference (Z =  2.26), indicating this structure’s prominence across linguistic groups.

Complex structures show considerably lower usage patterns. -*ed* clauses have lowest relative frequency among Russian (0.010), Turkish (0.011), and German (0.012) learners. All postnominal clausal modifiers display negative Z-scores across language backgrounds, suggesting their less frequent usage may be due to syntactic complexity or differences in language instruction emphasis.

Premodifying nouns show significant usage in Chinese (0.255) and Turkish (0.259) learners, potentially reflecting greater familiarity with this structure in these linguistic contexts. Z-scores reveal notable inter-group variations, with Arabic speakers showing highest preference (Z =  1.25) and Italian speakers lowest but still above average (Z =  0.82), suggesting linguistic or educational influences on modification strategies.

Postnominal phrasal modifiers maintain consistent patterns: prepositional phrases (of) range from 0.100 to 0.125, indicating their stable utility in expressing relationships. French and Russian speakers show particular preference for these structures (Z =  0.22 and 0.19 respectively), while prepositional phrases (other) display more variable Z-scores across languages, though generally above average.

Prepositional -*ing* clauses and noun complement clauses (that) show consistently low relative frequencies and negative Z-scores across all language backgrounds, suggesting their specialized use or steeper learning curve. Relative clauses, while less frequent than premodifying nouns and simple prepositional phrases, maintain moderate usage across languages, indicating they are more commonly used than other complex structures despite their complexity.

Examining the relative frequency and Z-score trends of noun phrase modification across learners with different L1s unveils complex patterns in how learners use this grammatical feature. Attributive adjectives exhibit consistently high relative frequencies and positive Z-scores across all L1s, confirming their ubiquity in learner language. Conversely, more complex structures like -*ed* and infinitive clauses demonstrate consistently low relative frequencies and negative Z-scores. This suggests that these forms are less frequently used, potentially due to their syntactic complexity or varying instructional emphasis across different L1s. Premodifying nouns show notable inter-group variations. Arabic speakers demonstrate the highest Z-score (1.25), while Italian speakers have the lowest positive Z-score (0.82). This variation may reflect linguistic or educational influences on noun phrase modification strategies. Postnominal phrasal modifiers, including prepositional phrases (of) and other types, generally have positive Z-scores, with French and Russian speakers showing a particular preference for prepositional phrases (of) (Z =  0.22 and 0.19, respectively). In contrast, prepositional -*ing* clauses consistently register negative Z-scores across all language backgrounds, reinforcing their infrequent use and suggesting their complexity poses a challenge for learners across linguistic groups. Lastly, relative clauses, while less frequent than premodifying nouns and simple prepositional phrases, display moderate usages across L2 learners of all first languages. It means that relative clauses, despite their complexity, are more commonly used than other complex structures like -*ed* clauses and infinitive clauses. Overall, these findings highlight the influence of L1-specific factors, such as linguistic typologies, on the acquisition and utilization of specific grammatical structures in learner language.

### Evolution of premodification and postmodification strategies across different proficiency levels

Table 4 presents a comprehensive analysis of the estimated effects of diverse noun modifiers across the spectrum of CEFR levels. The utilization of median values, accompanied by variability indicators such as the Median Absolute Deviation (MAD) and the 89% Highest Density Interval (HDI) confidence intervals, provides a detailed understanding of how different types of noun phrase modifications—premodification and postmodification—evolve as learners progress from B1 to C1 proficiency level.

**Table 4:** Estimated effects of noun modifiers across three CEFR levels.

Coefficients	Position	η	MAD	HDI 89%
alpha		23.868	2.441	18.70 - 30.51
Attributive adjectives at B1	Pre	0.554	0.075	0.373 - 0.729
Attributive adjectives at B2	Pre	0.481	0.068	0.320 - 0.642
Attributive adjectives at C1	Pre	0.434	0.055	0.304 - 0.566
Infinitive clauses at C1	Post	0.156	0.056	0.022 - 0.290
-*ing* clauses at C1	Post	0.146	0.057	0.013 - 0.279
Premodifying nouns at B1	Post	0.490	0.076	0.314 - 0.665
Premodifying nouns at B2	Post	0.397	0.069	0.236 - 0.560
Premodifying nouns at C1	Post	0.329	0.055	0.197 - 0.463
Prepositional phrases (of) at B1	Post	0.413	0.080	0.228 - 0.598
Prepositional phrases (of) at B2	Post	0.331	0.071	0.165 - 0.497
Prepositional phrases (of) at C1	Post	0.308	0.056	0.173 - 0.441
Prepositional phrases (other) at B1	Post	0.435	0.075	0.248 - 0.616
Prepositional phrases (other) at B2	Post	0.356	0.071	0.184 - 0.525
Prepositional phrases (other) at C1	Post	0.311	0.055	0.181 - 0.443
Prepositions + *ing* clauses at C1	Post	0.139	0.059	0.003 - 0.274
Relative clauses at B1	Post	0.343	0.080	0.156 - 0.529
Relative clauses at B2	Post	0.314	0.070	0.149 - 0.483
Relative clauses at C1	Post	0.354	0.056	0.221 - 0.485

Examining the use of attributive adjectives across proficiency levels reveals a gradual shift in their utilization. At the B1 level, there is a prominent reliance on these modifiers, with a median effect size of 0.554 (MAD: 0.075, CI [0.373 - 0.729]). However, a subtle decrease in effect size is observed at the B2 level (0.481, MAD: 0.068, CI [0.320 - 0.642]), suggesting a shift towards incorporating more varied structures as learners refine their linguistic skills. At the C1 level, the median effect size further diminishes to 0.434 (MAD: 0.055, CI [0.304 - 0.566]), reflecting a wider range of usage contexts for attributive adjectives in advanced language production.

At the C1 proficiency level, infinitive and -*ing* clauses demonstrate moderate effect sizes, with medians of 0.156 and 0.146, respectively. However, the broad confidence intervals indicate substantial individual variation in the frequency or application of these structures among learners. The use of premodifying nouns follows a distinct pattern across CEFR levels. At the B1 level, learners frequently employ these structures (η =  0.490, MAD: 0.076, CI [0.314 - 0.665]). However, this usage decreases at the B2 level (η =  0.397, MAD: 0.069, CI [0.236 - 0.560]) and further diminishes at the C1 level (η =  0.329, MAD: 0.055, CI [0.197 - 0.463]). This decline suggests a potential shift towards alternative noun modification strategies as learners advance in proficiency.

The use of prepositional phrases (of) varies across three CEFR levels, with B1 learners demonstrating a median effect size of 0.413 (MAD: 0.080, HDI [0.228 - 0.598]). This decreases at B2 (0.331, MAD: 0.071, HDI [0.165 - 0.497]) and C1 (0.308, MAD: 0.056, HDI [0.173 - 0.441]). Similarly, prepositional phrases (other) follow a comparable trend, with a median effect size of 0.435 (MAD: 0.075, HDI [0.248 - 0.616]) at B1, decreasing to 0.356 (MAD: 0.071, HDI [0.184 - 0.525]) at B2 and 0.311 (MAD: 0.055, HDI [0.181 - 0.443]) at C1.

C1 learners demonstrate a moderate effect size (η =  0.146) in their use of -*ing* clauses, with considerable variability observed (MAD: 0.057, HDI [0.013 - 0.279]). This indicates that while -*ing* clauses are employed at this advanced stage, their usage may vary across learners, as indicated by the range of values within the confidence interval. Relative clauses demonstrate a consistent presence across proficiency levels, albeit with slight fluctuations in effect size. The median effect size is substantial at the B1 level (0.343, MAD: 0.080, HDI [0.156 - 0.529]), indicating their fundamental role in early language production. This effect size slightly decreases at the B2 level (0.314, MAD: 0.070, HDI [0.149 - 0.483]), potentially reflecting a broadening of grammatical repertoire and the exploration of alternative structures. However, at the C1 level, the effect size rebounds slightly to 0.354 (MAD: 0.056, HDI [0.221 - 0.485]), suggesting a continued reliance on relative clauses for complex sentence constructions at higher proficiency levels.

## Discussion

### Highlighting the impact of L2 proficiency on L2 use of noun modifiers

The study’s analysis reveals distinct developmental patterns in the use of noun modifiers across three CEFR levels (B1-C1), highlighting that learners’ grammatical competence evolves with increasing proficiency. These patterns manifest differently across various modifier types, providing insights into the progression of L2 grammatical development.

Attributive adjectives emerge as the most consistently utilized modifier type across all proficiency levels, exhibiting both high relative frequencies and increasingly positive Z-scores as L2 proficiency advances. This dual indication suggests that learners not only maintain a strong reliance on these modifiers but also demonstrate growing sophistication in their usage, surpassing normative expectations for their respective proficiency levels. The increasing Z-scores particularly highlight learners’ expanding linguistic competence and growing comfort with these fundamental structures.

In contrast, premodifying nouns show a notable decline in both relative frequencies and Z-scores as proficiency increases. This pattern suggests a transitional phase in learners’ grammatical development, where they move away from basic, foundational structures toward more intricate linguistic constructions. The decrease in both usage frequency and normative comparison underscores a deliberate shift in learners’ grammatical preferences, indicating a natural progression toward more sophisticated linguistic expression.

Postnominal phrasal modifiers demonstrate a more nuanced development pattern. While their frequency shows a slight increase across rising proficiency levels, their Z-scores maintain relative stability. This pattern indicates that learners gradually incorporate these structures into their language use in alignment with expected norms, suggesting a measured and systematic integration of these modifiers without significant deviation from typical usage patterns at each level.

The development pattern becomes particularly interesting when examining complex clausal modifiers. These structures, including -ed clauses and infinitive clauses, consistently show low relative frequencies and negative Z-scores across all proficiency levels, highlighting the persistent challenges learners face with these more sophisticated modifications. Specifically, -ed clauses exhibit a decrease in relative frequency from B1 to B2, followed by a modest increase at C1, while maintaining negative Z-scores throughout the progression. Similarly, infinitive clauses display consistently low relative frequencies and negative Z-scores, indicating that these complex structures remain challenging even at higher proficiency levels.

Relative clauses, however, present a distinctive developmental trajectory. Unlike other complex clausal modifiers, they show an increasing trend in both relative frequency and Z-scores from B1 to C1. This progressive improvement suggests that while learners initially struggle with relative clauses, they gradually develop mastery of these structures as their proficiency improves. This pattern indicates that relative clauses, though initially challenging, become more accessible and manageable for learners at higher proficiency levels.

The overall findings suggest that L2 learners across different proficiency levels demonstrate a stronger tendency toward using premodifiers compared to postmodifiers, aligning with previous research findings by Biber et al. [8], Parkinson and Musgrave [13], and Wang and Beckett [16]. This preference indicates that modification in L2 acquisition emerges not as an independent grammatical construct but rather as an integrated component of broader grammatical development. The successful deployment of different modifier types requires mastery of various grammatical components - from basic adjective-noun agreement in attributive adjectives to complex clause structures and relative pronoun usage in relative clauses.

This developmental pattern suggests a hierarchical progression in L2 grammatical acquisition, where learners first master simpler modification structures before gradually incorporating more complex ones into their linguistic repertoire. The consistent challenges with certain complex clausal modifiers, even at higher proficiency levels, underscore the need for targeted pedagogical intervention in these areas, particularly in supporting learners’ transition from basic to more sophisticated modification strategies.

### Downplaying the influence of L1 background on L2 use of noun modifiers

The study reveals a complex relationship between learners’ L1 background and their use of noun modifiers in English, with the overall influence of L1 being more limited than traditionally assumed. This finding emerges from a detailed analysis of modifier usage patterns across different L1 groups and proficiency levels.

Across all L1 groups, attributive adjectives consistently demonstrate high relative frequencies and positive Z-scores, suggesting a universal preference for this modifier type. This consistency indicates that attributive adjectives function as fundamental and readily accessible components of English grammar, transcending linguistic backgrounds. This universality likely stems from their direct and straightforward role in noun modification. Regression analysis reveals an interesting developmental trajectory: while B1 level learners show strong reliance on these modifiers, as evidenced by higher effect sizes, this dependence gradually decreases as learners progress to C1 level, indicating a shift toward more varied and sophisticated modifier usage.

The regression analysis further elucidates several key developmental trends that appear consistent across L1 groups. A notable decline in the use of premodifying nouns and prepositional phrases from B1 to C1 signifies progression toward more complex grammatical structures. Additionally, increased effect sizes for postnominal clausal modifiers, such as infinitive and -ing clauses, at higher proficiency levels demonstrate growing comfort with sophisticated grammatical forms. These patterns suggest that variations in noun phrase modification align more closely with proficiency levels than with L1 backgrounds, challenging the prevailing notion that L1 invariably plays a critical role in language acquisition.

However, it would be inadvisable to entirely overlook the potential influence of first language on the use of noun modifiers in second language acquisition. The influence of learners’ first languages becomes particularly visible in complex grammatical structures. For instance, the use of premodifying nouns and postnominal phrasal modifiers shows variable trends among different L1 groups, as indicated by both relative frequencies and Z-scores. Some L1 groups, such as Arabic and Turkish speakers, exhibit higher relative frequencies and more positive Z-scores for premodifying nouns compared to other groups. This might suggest a linguistic or educational predisposition towards using these structures more frequently, reflecting syntactic parallels or pedagogical emphasis in their native languages.

Complex clausal modifiers such as -*ed* and -*ing* clauses generally demonstrate lower relative frequencies and often negative Z-scores, though not universally across all first languages. This variability suggests a shared challenge with these structures, albeit with some differences in the difficulty level, which may be influenced by learners’ linguistic backgrounds. In this sense, this study shares the view of previous studies that L2 use of noun modifiers is prone to the influence of the learners’ first languages [23,25,26].

The impact of L1 on noun phrase modification appears most pronounced in the early stages of language acquisition, where learners’ native linguistic frameworks heavily influence their initial modification strategies. This study’s relative frequency analysis reveals distinct L1-based patterns in the usage of specific noun modifiers, such as the higher frequency of attributive adjectives by Arabic speakers compared to Turkish speakers. However, as learners attain higher proficiency levels, these distinct impacts of L1 wane, leading to a more uniform use of NP structures that align closely with target language norms. While frequency analyses indicate potential L1 transfer effects, particularly in the early stages of acquisition, the regression analysis indicates a convergence towards target language norms, regardless of L1 backgrounds.

### Revealing the natural evolution of L2 noun premodification and postmodification strategies

The analysis of how premodification and postmodification strategies evolve with increasing L2 proficiency reveals systematic developmental patterns that align with and extend previous research findings. These patterns provide important insights into how learners progress from basic to more sophisticated modification strategies as their language skills develop.

The developmental trajectory of premodification strategies shows a clear pattern of change. As learners advance in proficiency, they demonstrate decreasing reliance on premodifying nouns, suggesting a shift away from simpler modification structures. This finding aligns with Parkinson and Musgrave’s [13] observation that less proficient learners tend to overuse simpler modification structures, while more advanced learners diversify their modification strategies. The gradual decrease in premodification use likely reflects learners’ growing linguistic maturity and their increasing ability to employ alternative modification strategies, supporting Wang and Beckett’s [16] findings about the evolution of modification patterns in L2 writing.

The development of postmodification abilities presents a more complex picture. The slight but consistent increase in postnominal phrasal modifiers, particularly prepositional phrases, across proficiency levels suggests a gradual expansion of learners’ grammatical repertoire. This trend supports Biber et al.’s [8] developmental stages theory, which proposes that learners progressively incorporate more sophisticated modification patterns as their proficiency increases. The findings particularly reinforce their observation that prepositional phrases with specific meanings emerge before those with more abstract meanings, indicating a staged development in postmodification abilities.

More complex postmodification structures, such as infinitive and -*ing* clauses, show interesting developmental patterns that support previous research findings. The increasing use of these structures at higher proficiency levels aligns with Liu and Li’s [14] observations about the gradual development of complex noun phrase structures in L2 writing. However, the persistent challenges learners face with these structures, even at advanced levels, echo Ansarifar et al.’s [17] findings about the differences between L2 learners and expert writers in handling complex noun modifications.

The development of relative clause usage presents a particularly noteworthy pattern. While these structures remain challenging across all proficiency levels, learners show gradual improvement in their ability to use them effectively. This finding supports Díez-Bedmar and Pérez-Paredes’ [22] research with young Spanish learners, which found that relative clause usage increases with proficiency development. The pattern also aligns with Sarte and Gnevsheva’s [23] observation that higher proficiency learners demonstrate greater facility with complex modification structures.

These findings collectively suggest a developmental sequence that moves from simpler to more complex modification strategies, supporting Biber et al.’s [8] proposed stages of grammatical development. As learners progress, they gradually transition from heavy reliance on basic premodification patterns toward more balanced and sophisticated modification strategies. This evolution reflects Lan and Sun’s [18] as well as Lan et al.’s [19] findings that higher writing proficiency correlates with more diverse and sophisticated noun modification patterns. The parallel trends of decreasing premodification and increasing complex postmodification use suggest a fundamental evolution in learners’ grammatical capabilities, supporting Wang and Slater’s [15] observations about the relationship between proficiency level and syntactic complexity in L2 writing.

## Conclusion

### Major findings

The analysis of noun modifier usage in the EFCAMDAT corpus reveals that proficiency level, rather than first language background, determines grammatical complexity in second language development. The Bayesian analysis of learner data yields three significant findings about L2 grammatical development.

The first finding concerns the consistency of developmental patterns across different proficiency levels. Learners at all proficiency levels show a strong command of attributive adjectives, while complex structures like -*ed* clauses and infinitive clauses remain challenging even at advanced levels. The regression analysis indicates that this pattern holds true regardless of first language background, suggesting universal pathways in the development of grammatical complexity.

The second finding challenges previous assumptions about first language influence on grammatical development. While early-stage learning exhibits some L1-specific patterns, particularly in the use of premodifying nouns and basic postmodification structures, these differences diminish significantly as proficiency increases. The statistical evidence indicates convergence toward similar patterns of grammatical usage among advanced learners from different L1 backgrounds.

The third finding establishes proficiency level as the primary factor in grammatical development. The data show systematic progression from simple to complex modification structures as proficiency increases. This development manifests through decreased reliance on basic premodifiers and increased mastery of sophisticated postmodification structures, reflecting the natural evolution of grammatical competence.

The Bayesian methodology employed in this study allowed for rigorous testing of theoretical assumptions about language development. The incorporation of prior knowledge about grammatical development into the analysis framework enabled systematic evaluation of L1 influence hypotheses. The results demonstrate that while L1 background may influence initial learning stages, its effect becomes minimal compared to the role of overall language proficiency.

The effectiveness of this methodological approach is further validated by recent studies employing similar analytical frameworks to L2 writing analysis. Alzahrani [32], using Kolmogorov complexity measures across ten L1 backgrounds, demonstrated distinct syntactic complexity patterns in L2 English writing that align with the observed variations in noun modifier usage. The Kolmogorov complexity analysis, being language-general and unsupervised, provided complementary evidence for the developmental patterns observed across proficiency levels.

In a related study, Bozdağ et al. [33] employed Bayesian probabilistic analysis to examine modal verb usage among Turkish and Chinese learners, revealing that L1 influence becomes less pronounced when controlling for contextual factors. This finding corroborates the observation that L1-specific patterns in noun modifier usage diminish as proficiency increases. Their discovery that context-specific analysis reveals subtle variations enhances the understanding of how L1 background influences grammatical feature usage in L2 writing.

The convergence of evidence from these studies using different methodological approaches—Kolmogorov complexity measures and Bayesian probabilistic analysis—strengthens the conclusions about the relationship between L1 background and grammatical development in L2 writing. These studies not only validate the current methodological approach but also suggest that the influence of L1 on grammatical structures operates within a complex interplay of factors including proficiency level, topic, and context. This multi-faceted understanding reinforces the finding that language instruction should focus on overall proficiency development while remaining cognizant of how contextual factors may influence grammatical development.

These findings advance the understanding of second language acquisition in two ways. Empirically, they establish that grammatical development follows similar trajectories across different first languages. Methodologically, they demonstrate the effectiveness of Bayesian analysis in testing theoretical assumptions about language learning. This improved understanding suggests that language instruction should prioritize overall proficiency development rather than focusing on presumed L1-specific challenges.

### Pedagogical implications

The study’s findings address three significant gaps in existing research on L2 noun modifier usage, each with distinct pedagogical implications. Regarding the first research gap—the applicability of findings beyond academic writing—this study demonstrates that the challenges with complex noun phrase structures persist in general writing contexts. The consistent underuse of postnominal clausal modifiers across proficiency levels indicates that these difficulties are not confined to academic genres. This finding suggests the need for explicit instruction in complex noun phrase structures across all writing contexts, not just academic writing.

The second research gap—understanding how L1 influence changes with proficiency—is addressed through the finding that L1 effects diminish as proficiency increases. This insight has important implications for pedagogical practice. While early-stage instruction might benefit from awareness of L1-specific challenges, advanced instruction should focus on universal aspects of grammatical development. The evidence suggests that teaching strategies should evolve with learner proficiency, moving from L1-sensitive approaches at lower levels to more generalized instruction at higher levels.

Addressing the third gap—determining the relative impact of L2 proficiency versus L1 background—the study reveals proficiency level as the primary factor in grammatical development. This finding aligns with observations by Carrió Pastor [25] and Guillerit [9] about the universal challenges of complex noun phrases, which affect both L2 learners and native speakers. The pedagogical implication is that instruction should prioritize systematic development of grammatical complexity rather than focusing on L1-specific interventions.

These findings collectively suggest a need for targeted pedagogical approaches. First, explicit instruction in complex noun phrase structures should begin early and continue throughout all proficiency levels. Second, teaching methods should adapt to learners’ developing proficiency, gradually introducing more complex structures while maintaining focus on challenging areas such as postnominal clausal modifiers. Finally, instruction should emphasize the universal aspects of grammatical development, particularly at advanced levels where L1 influence becomes less significant.

### Limitations and suggestions for future studies

It is necessary to point out that this study has several methodological limitations. First, there is a potential risk in applying a script developed for extracting noun phrase modifications from a grammatically accurate corpus to investigate a learner corpus. While it demonstrated high accuracy within a well-organized corpus containing properly constructed sentences, its effectiveness in a learner corpus—where sentences may be less formal and potentially contain errors—remains uncertain. Second, the Bayesian regression approach employed in this study, while robust, presents inherent limitations. The results may be sensitive to the choice of informative priors based on expected noun modifier development patterns, potentially biasing findings toward anticipated trajectories. Additionally, the negative binomial regression model assumes specific distribution patterns that may not fully capture the complexity of language development. Third, interpretation of L1 effects on second language acquisition depends on the selection of confidence interval. This study’s finding that there was no significant impact of learners’ first languages on their use of noun modifiers was based on an 89% confidence interval recommended by Kruschke [34] who argued that this range offered greater stability compared to higher intervals. However, selecting a different confidence interval might produce varying results. Fourth, there is a latent problem with relying on the CEFR guidelines, which presuppose a linear progression in language proficiency. This model assumes that mastery of grammatical structures at one level facilitates advancement to the next. However, since the study did not assess the grammatical accuracy of the phrases—such as the correct order of adjectives in attributive modifiers—the findings reflected the prevalence of certain structures rather than the learners’ accurate use of them.

Future research should address these limitations through several approaches. First, the extraction script should be enhanced to better accommodate the diverse and potentially erroneous structures inherent in learner corpora. This improvement should include the ability to assess the grammatical correctness of noun phrase modifications and extend the analysis beyond mere frequency counts to evaluate learners’ actual language proficiency. Second, alternative statistical approaches could be compared with Bayesian results to validate findings across different methodological frameworks. Third, subsequent studies should investigate the influence of learners’ first languages more comprehensively, potentially employing alternative confidence interval ranges to achieve a more nuanced understanding of L1 effects on L2 acquisition. Lastly, future studies should prioritize the authenticity and correctness of language use over mere prevalence to provide a clearer assessment of learners’ mastery of grammatical structures. Adopting this comprehensive approach will yield more insightful and applicable findings for language education and curriculum development.
